# Sedation in Gastrointestinal Endoscopy: From Drug-Centered Protocols to Personalized, Technology-Supported Pathways: A Narrative Review

**DOI:** 10.3390/jcm15114281

**Published:** 2026-06-01

**Authors:** Giuliano Francesco Bonura, Paola Soriani, Noemi Gualandi, Pablo Cortegoso Valdivia, Tommaso Gabbani, Arianna Parrella, Anastasios Koulaouzidis, Mauro Manno

**Affiliations:** 1 Gastroenterology and Digestive Endoscopy Unit, Azienda USL di Modena, 41121 Modena, Italy; 2 Gastroenterology and Endoscopy Unit, University Hospital of Parma, 43126 Parma, Italy; 3 Department of Clinical Research, University of Southern Denmark, 5230 Odense, Denmark; 4 Surgical Research Unit, Odense University Hospital, 5000 Odense, Denmark; 5 Department of Medicine, Odense University Hospital, 5700 Svendborg, Denmark; 6 Department of Social Medicine and Public Health, Pomeranian Medical University, 71-210 Szczecin, Poland

**Keywords:** gastrointestinal endoscopy, sedation, propofol, patient safety, intraprocedural monitoring, sedation training, artificial intelligence

## Abstract

**Background/Objectives**: Sedation is a fundamental component of gastrointestinal endoscopy, improving patient comfort, procedural quality, and overall satisfaction. However, traditional drug-centered sedation models are increasingly challenged by rising procedural volumes, aging populations, and limited anesthesiology resources. The aim of this narrative review is to provide an integrated overview of evolving pharmacological agents, monitoring strategies, organizational models, and future directions toward personalized, technology-supported sedation pathways. **Methods**: A structured literature search was conducted across PubMed/MEDLINE, Scopus, and Web of Science for studies published between January 2010 and December 2025. Relevant guidelines, randomized controlled trials, meta-analyses, and large observational studies were included. Evidence was synthesized qualitatively, emphasizing clinical applicability and real-world relevance. **Results**: Propofol remains the most widely used sedative agent due to its rapid onset and recovery profile, although its narrow therapeutic window and lack of antagonist limit its safety in high-risk patients. Emerging agents such as remimazolam and ciprofol demonstrate comparable efficacy with improved respiratory and hemodynamic safety profiles, particularly in elderly populations. Adjunctive strategies, including procedure-specific approaches such as spinal anesthesia, may further optimize sedation. Advanced monitoring tools, such as capnography, bispectral index, and high-flow nasal cannula, show potential in enhancing safety, especially in selected high-risk groups. Structured training programs and standardized discharge criteria are essential for ensuring quality and safety. **Conclusions**: Sedation in gastrointestinal endoscopy is transitioning from a standardized, drug-centered approach to a personalized, risk-adapted, and technology-supported model. Integration of novel pharmacological agents, advanced monitoring, and structured training will be key to improving patient safety, procedural efficiency, and healthcare sustainability.

## 1. Introduction

Sedation has emerged as a cornerstone of modern gastrointestinal (GI) endoscopy, with extensive evidence supporting its role in improving procedural quality, patient comfort, and overall satisfaction for both patients and endoscopists [1,2,3,4,5]. Over the past decades, sedation practices in GI endoscopy have evolved substantially. This evolution has been driven by the increasing complexity and duration of endoscopic procedures, the continuous growth in procedural volumes [6,7], and a heightened focus on patient safety and quality of care. At the same time, demographic changes, including population aging and a rising prevalence of comorbidities, have led to a growing proportion of high-risk and frail patients undergoing endoscopic interventions [8,9]. These trends have exposed the limitations of traditional sedation models, particularly in the context of limited availability of anesthesiology resources in many healthcare systems [10]. Therefore, alternative sedation strategies have been progressively developed and implemented to address these clinical and organizational constraints. Traditional approaches based on conventional sedative regimens are increasingly being complemented or replaced by innovative pharmacological solutions, redesigned organizational models, and advanced monitoring technologies [11]. In this context, comprehensive training represents a key element to guarantee the safe and effective implementation of these evolving sedation models, thereby ensuring procedural efficacy, patient safety, and system sustainability [12].

Despite these advances, current sedation paradigms in GI endoscopy remain largely drug-centered and insufficiently adapted to contemporary clinical and organizational demands. The widespread reliance on propofol-based deep sedation, often dependent on anesthesiology resources, is increasingly misaligned with the growing procedural volume, the aging endoscopic population, and the rising prevalence of frailty and comorbidity. In many healthcare systems, this model is becoming operationally unsustainable and clinically suboptimal. Consequently, there is an urgent need to move beyond a ‘one-size-fits-all’ approach toward sedation pathways that are individualized, risk-adapted, and supported by organizational innovation and advanced monitoring technologies [13]. This narrative review aims to provide an integrated overview of emerging pharmacological agents, evolving organizational models, advanced monitoring technologies, training strategies, and sustainability considerations that are collectively reshaping sedation pathways in contemporary GI endoscopy.

## 2. Materials and Methods

This narrative review was conducted using a structured and comprehensive literature search strategy aimed at summarizing and critically appraising contemporary evidence on sedation practices in GI endoscopy.

### 2.1. Literature Search Strategy

A systematic search of PubMed/MEDLINE, Scopus, and Web of Science databases was performed to identify relevant articles published between January 2010 and December 2025. The time frame was selected to capture both established sedation practices and recent pharmacological and technological innovations. The search strategy combined Medical Subject Headings (MeSH) and free-text terms using Boolean operators (“AND”, “OR”). Key terms included: “gastrointestinal endoscopy”, “sedation”, “propofol”, “remimazolam”, “ciprofol”, “non-anesthesiologist-administered sedation”, “patient safety”, “monitoring”, “capnography”, “training”, “artificial intelligence”. In addition, the reference lists of selected articles, relevant guidelines, and position statements were manually screened to identify further pertinent studies not retrieved through the database search.

### 2.2. Study Selection and Eligibility Criteria

Studies were selected based on clinical relevance, methodological robustness, and real-world applicability, with particular emphasis on evidence directly informing clinical decision-making and organizational aspects of sedation in GI endoscopy. Inclusion criteria were international guidelines and consensus statements, randomized controlled trials (RCT), meta-analyses and systematic reviews, large prospective or retrospective observational studies, and position statements from major scientific societies. Exclusion criteria were studies focusing exclusively on pediatric populations (<18 years), studies not involving GI endoscopic procedures, case reports, small case series, conference abstracts, and non-English publications.

When multiple studies addressed the same topic, priority was given to the most recent, methodologically rigorous, and comprehensive evidence, particularly meta-analyses and large multicenter trials.

### 2.3. Data Synthesis and Scope

Given the narrative nature of this review, no formal systematic review methodology was applied, and quantitative data pooling or risk-of-bias assessment was not performed. Instead, evidence was synthesized qualitatively to provide a balanced and critical overview of evolving pharmacological agents, intraprocedural monitoring strategies, training models, postprocedural care, and emerging future directions, including artificial intelligence (AI) and sustainability considerations. The review intentionally integrates pharmacological, technological, organizational, and educational perspectives, reflecting the multidimensional nature of sedation practice in contemporary GI endoscopy.

## 3. Pharmacological Agents

Current international guidelines recommend offering sedation to all patients undergoing GI endoscopic procedures [14,15,16,17,18]. In general, the type and depth of sedation should generally be tailored based on the patient’s risk assessment and the procedural complexity [15]. However, unsedated endoscopy may be considered according to the patient’s risk profile and preferences [18,19]. Selected procedures can indeed be performed without sedation, with acceptable patient tolerance, preserving the quality of endoscopic procedure [20,21].

Traditionally, sedation for GI endoscopy has relied on benzodiazepines and opioids, administered either alone or in combination [22]. Midazolam has long been the most widely used benzodiazepine due to its rapid onset and relatively short duration of action [23]. Nevertheless, the introduction of newer ultra–short-acting agents, including remimazolam, is likely to progressively reduce its use [24,25]. Despite increasing interest in newer agents, midazolam remains clinically relevant, particularly for conscious and moderate sedation and in combination with opioids. Its advantages include familiarity, anxiolysis, amnesia, and reversibility with flumazenil. However, its use requires caution because of interindividual variability, prolonged recovery, accumulation in elderly or frail patients, and dose-dependent respiratory depression, especially when combined with opioids. Therefore, midazolam remains useful in selected settings but should be incorporated into risk-adapted sedation strategies rather than applied as a default regimen. Among opioids, fentanyl has long been the most used agent and is still widely utilized in current practice, especially as an adjunct to propofol or benzodiazepines, owing to its analgesic efficacy and rapid onset of action [17]. However, its use requires careful titration because of the risk of dose-dependent respiratory depression, particularly when combined with other sedative agents [23].

Advances in pharmacology have led to the development and increasing adoption of newer sedative and anesthetic agents for GI endoscopy. These drugs are designed to address some of the limitations of traditional sedation strategies. This section focuses on pharmacological agents with the most robust evidence and established applicability to routine GI endoscopy, while emerging combinations primarily investigated in anesthesiology settings are discussed only where directly relevant. Table 1 summarizes the main characteristics of the pharmacological agents discussed in this review.

### 3.1. Propofol

Propofol use has increased substantially over recent decades, and a recent European Society of Gastrointestinal Endoscopy (ESGE) survey identified it as the most commonly used sedative agent in GI endoscopy [13].

Propofol produces dose-dependent sedation through potentiation of γ-aminobutyric acid type A receptor activity. Compared with traditional benzodiazepine–opioid regimens, propofol-based sedation is associated with faster recovery, improved procedural efficiency, and higher patient satisfaction, while maintaining a comparable overall safety profile when appropriately administered [26].

However, propofol has a narrow therapeutic window and no specific antagonist. Dose-dependent respiratory depression and hypotension remain its main limitations, particularly in elderly and high-risk patients. For this reason, careful patient selection, continuous monitoring, and structured training are essential [27].

Although propofol remains the reference sedative agent in GI endoscopy, emerging evidence suggests that newer agents, such as remimazolam and ciprofol, may offer comparable efficacy with potential safety advantages in selected patient populations [24,28]. Consequently, propofol is increasingly being integrated into individualized, risk-adapted sedation strategies rather than used as a universal solution.

### 3.2. Remimazolam

Remimazolam is a novel ultra-short-acting, water-soluble benzodiazepine that, similarly to midazolam, acts as a positive allosteric modulator of the γ-aminobutyric acid type A receptor, thereby exerting anxiolytic and sedative effects. Since its approval for clinical use, several RCTs have demonstrated that remimazolam is non-inferior to propofol in terms of sedation success, while offering a more favorable safety profile, particularly in elderly and high-risk patients [29,30,31,32,33,34,35,36]. A multicenter RCT further showed that the combination of remimazolam and propofol was associated with improved safety compared with propofol alone, and superior efficacy and patient satisfaction compared with remimazolam monotherapy [37]. Moreover, in a recent multicenter RCT, Choe et al. demonstrated the superiority of remimazolam over propofol in patients undergoing endoscopic ultrasound [38]. In addition, five meta-analyses, three of which specifically focused on elderly populations, have consistently reported comparable efficacy between remimazolam and propofol, with a superior safety profile for remimazolam, particularly regarding respiratory and hemodynamic adverse events [39,40,41,42,43]. Finally, a meta-analysis including nearly 1000 patients undergoing endoscopic retrograde cholangiopancreatography (ERCP) confirmed the favorable safety profile of remimazolam in this setting, although a higher incidence of tachycardia and procedure-related body movements was observed [44]. The main characteristics of the included meta-analyses are summarized in Table 2. Although the current body of evidence is promising, it should be interpreted with caution, as it is still largely based on RCTs and meta-analyses, with limited real-world data available. At present, remimazolam may represent a potentially useful option for sedation, particularly in elderly and high-risk populations; however, further real-life studies and broader clinical experience are needed before drawing definitive conclusions or considering its routine use as an alternative to propofol in all patients.

### 3.3. Ciprofol

Ciprofol is a novel 2,6-disubstituted alkylphenol anesthetic, sharing structural similarities and a comparable mechanism of action with propofol [45]. Current clinical experience with ciprofol is largely derived from studies conducted in China, and its regulatory approval and availability remain limited in many regions. In the past year, several meta-analyses have compared ciprofol with propofol, demonstrating similar efficacy with potential advantages in terms of adverse event profiles [46,47,48,49,50,51]. Additionally, Wu et al. conducted a systematic review and meta-analysis comparing ciprofol and propofol in patients undergoing ERCP. Across seven RCTs involving 1264 participants, ciprofol was associated with a significantly lower incidence of bradycardia, respiratory depression, hypoxemia, and injection pain compared with propofol [52]. The main characteristics of the included meta-analyses are summarized in Table 3.

Taken together, although the available evidence is encouraging, it should be interpreted with caution due to the limited geographic distribution of studies and the predominance of data from RCTs. At present, ciprofol may represent a promising alternative for sedation in GI endoscopy; however, further large-scale, high-quality and real-world studies are needed before it can be considered a fully established alternative to propofol.

### 3.4. Dexmedetomidine

Dexmedetomidine, a relatively new α2-adrenergic receptor agonist, provides sedation, analgesia, and anxiolysis without causing respiratory depression [23]. Although when used alone it may not provide sufficient sedation and is associated with a high incidence of bradycardia [53], some RCTs investigated its role as an adjuvant in combination with propofol in different GI endoscopic procedures both diagnostic and therapeutic [54,55,56]. These studies suggest that dexmedetomidine can reduce propofol requirements and consequently lower the risk of propofol-related adverse events, such as respiratory depression. Nevertheless, the frequent occurrence of bradycardia and prolonged discharge times may limit its routine use, as highlighted in a recent meta-analysis [57]. Overall, dexmedetomidine appears best suited as an adjunct to reduce propofol requirements rather than as a primary sedative agent in GI endoscopy.

### 3.5. Spinal Anesthesia

Spinal anesthesia (SA) is a well-established neuraxial technique characterized by a rapid onset of profound sensory and motor blockade following the intrathecal administration of local anesthetics [58]. Advanced therapeutic endoscopic procedures, including complex recto-sigmoid resections, are often technically demanding and prolonged, requiring sustained patient immobility. These interventions are frequently associated with significant intra- and post-procedural abdominal discomfort, which may necessitate high doses of sedative agents and consequently increase the risk of sedation-related adverse events [59]. Evidence from a pilot study suggests that SA is both safe and effective in patients undergoing endoscopic submucosal dissection of large recto-sigmoid lesions, with no need for conversion to deep sedation. In addition, only minimal doses of systemic fentanyl and/or midazolam were required in approximately half of the cases. SA represents a procedure-specific anesthetic alternative rather than a conventional sedation strategy and may be relevant in selected advanced therapeutic endoscopic interventions, particularly in elderly or frail patients and in procedures involving lesions extending to the anal canal [60].

## 4. Intraprocedural Monitoring

While conventional monitoring remains the standard of care during endoscopic procedures (i.e., non-invasive blood pressure measurement, heart rate and electrocardiographic monitoring, continuous pulse oximetry, and supplemental oxygen administration), increasing attention has been directed toward the development and implementation of novel monitoring and oxygenation strategies. These emerging technologies aim to further improve patient safety, with particular emphasis on the early detection and prevention of hypoxemia and sedation-related adverse events during GI endoscopy.

The clinical utility of advanced monitoring and oxygenation strategies should be interpreted in the context of patient-related risk factors, procedural complexity, and sedation depth. While these technologies have shown potential benefits in selected settings, their impact is not uniform across all patient populations, and their routine use should be individualized according to clinical and organizational requirements. Furthermore, it is important to distinguish between surrogate endpoints (such as oxygen saturation, end-tidal CO_2_) and clinically relevant outcomes (such as hypoxemia requiring intervention, airway management, or serious adverse events), as the evidence for advanced monitoring technologies often differs between these two domains.

### 4.1. Capnography

Since 2015, when the use of capnography monitoring was recommended for consideration in high-risk patients, during deep sedation with propofol, and in long-lasting procedures [17], it has been extensively studied in both diagnostic procedure and operative procedures, such as endoscopic retrograde cholangiopancreatography, endoscopic ultrasound, and percutaneous endoscopic gastrostomy placement, with particular attention to obese patients [61,62,63]. Moreover, a recent ESGE survey highlighted that capnography monitoring has increasingly been used for deeper sedation than during moderate sedation [13].

**Figure 1:**
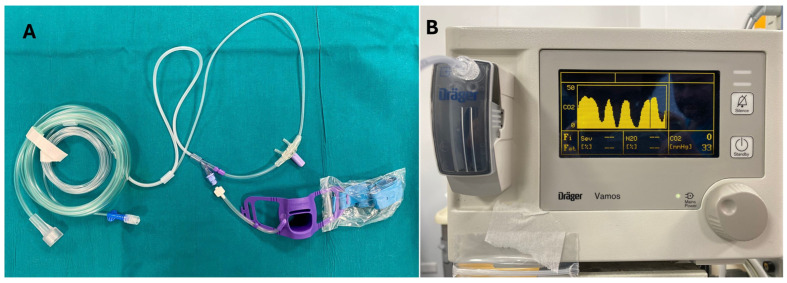
The device used to monitor capnometry (**A**); the monitor displays the end-tidal carbon dioxide (**B**).

The use of capnography monitoring (Figure 1) has been associated with a lower incidence of sedation-related adverse events and a reduced need for airway interventions, primarily due to its ability to identify respiratory insufficiency and hypoventilation earlier than peripheral oxygen saturation monitoring [64,65]. While a meta-analysis suggested that capnography monitoring should be routinely implemented because of its favorable impact on patient safety [66], a recent randomized controlled trial by Takimoto et al. reported no significant reduction in hypoxemia among healthy, low-risk, non-obese patients, highlighting the need for further studies to clarify the role of capnography in higher-risk populations undergoing deep sedation [62]. Finally, a recent multicenter, randomized, interventional superiority trial focusing on elderly patients aged 65–79 years showed that capnography monitoring was associated with a significant reduction in hypoxemia compared with standard monitoring [67].

These findings suggest that capnography may offer greater clinical benefit in selected high-risk populations, including older patients and those undergoing complex procedures.

### 4.2. Bispectral Index

**Figure 2:**
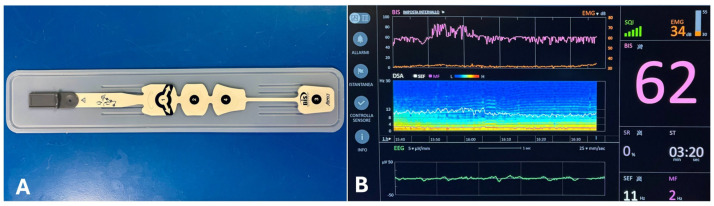
The device used to monitor the bispectral index (**A**); the monitor displays the bispectral index (**B**).

The Bispectral Index (BIS) is a dimensionless numerical scale derived from electroencephalographic signal analysis that provides an objective assessment of sedation depth by quantifying the level of consciousness (Figure 2). Although BIS monitoring is widely used in anesthesia practice, its routine application in GI endoscopy remains controversial. The principal benefit associated with BIS-guided sedation is a reduction in propofol consumption, particularly reflected by lower mean infusion rates [68], with elderly patients requiring significantly lower doses when managed using target-controlled infusion combined with BIS monitoring compared with younger individuals [69]. A meta-analysis confirmed that BIS monitoring reduces propofol consumption and may prevent unnecessary drug administration while maintaining adequate sedation; however, it did not demonstrate significant benefits in terms of recovery time, procedure duration, adverse events, or satisfaction [69]. Similarly, another recent meta-analysis reported a reduced incidence of intraprocedural hypoxia in the BIS group, but found no meaningful differences in procedural outcomes, recovery time, hemodynamic parameters, or satisfaction scores [70].

Overall, while BIS monitoring appears to optimize propofol administration, the available evidence has not consistently demonstrated sufficient evidence to support its routine use across all clinical settings. Its potential role may be more relevant in selected settings where precise titration of sedative agents is required.

### 4.3. High-Flow Nasal Cannula

**Figure 3:**
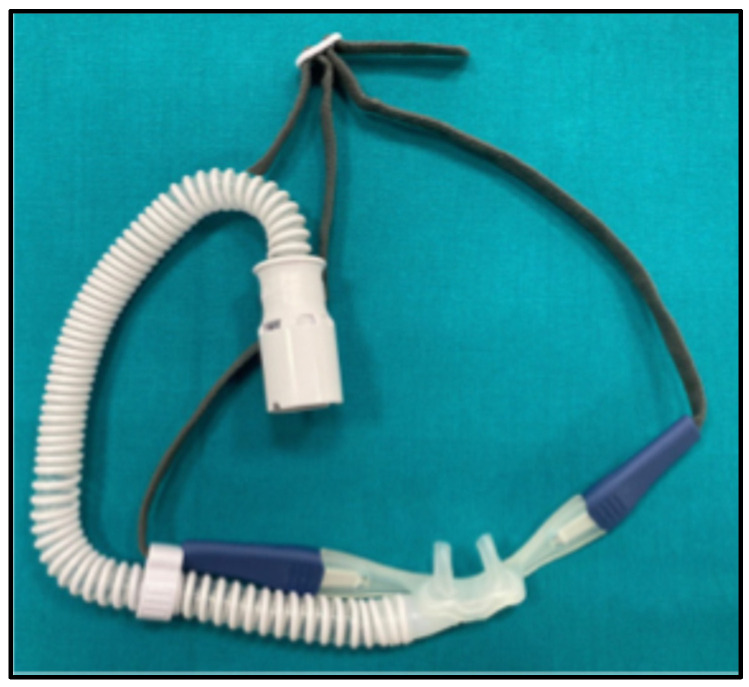
High-flow nasal cannula.

High-flow nasal cannula (HFNC) therapy represents a noninvasive oxygenation strategy that delivers heated and humidified oxygen–air mixtures at high flow rates, thereby improving oxygen delivery and respiratory support during sedation (Figure 3). Four recent systematic reviews and meta-analyses demonstrated that HFNC use is associated with a significant reduction in the incidence of hypoxemia and a decreased need for airway interventions, particularly among patients at low risk for hypoxemia; conversely no differences were observed in patients at high risk for hypoxemia [71,72,73,74]. Wang et al., in a recent multicenter, randomized parallel group study on 1000 adult obese patients (body mass index ≥ 28), demonstrated that oxygen delivery via HFNC reduced the incidence of hypoxemia, subclinical respiratory depression, and severe hypoxemia [75].

Nevertheless, the interpretation of these findings is limited by considerable heterogeneity across studies, differences in patient populations, procedural settings, and sedation strategies, as well as an overall moderate-to-high risk of bias. Accordingly, the available evidence does not support routine HFNC use in all patients undergoing procedural sedation, although it may be considered selectively according to patient risk profile, procedural characteristics, and planned depth of sedation.

## 5. Postprocedural Phase

According to international guidelines, patients undergoing GI endoscopy should receive structured postprocedural monitoring by trained personnel capable of promptly recognizing and managing procedure-related adverse events. Monitoring should be continued until the patient has fully returned to their baseline clinical status [15,16,17,18]. Despite these recommendations, evidence regarding the optimal type, duration, and intensity of postprocedural monitoring remains limited.

### 5.1. Discharge Criteria

The increasing emphasis on outpatient GI endoscopy has placed postprocedural discharge processes at the center of quality assessment, highlighting the need for standardized and reproducible discharge criteria to ensure patient safety. Several discharge scoring systems have been proposed and validated, demonstrating the ability to facilitate both safe and earlier discharge. Among these, the Aldrete score and the modified Post-Anesthetic Discharge Scoring System (mPADDS) are the most widely used and reliable tools [76]. A randomized controlled trial demonstrated that use of the mPADDS significantly reduced recovery time without increasing the incidence of adverse events [77]. Furthermore, a propensity score-matched study by Yamaguchi et al. comparing the modified Aldrete score with the mPADDS showed that while the modified Aldrete score allowed earlier discharge, it was associated with a higher prevalence of residual drowsiness at discharge [78]. Finally, two prospective observational studies confirmed that both the Aldrete score and the mPADDS enable earlier and safe discharge when compared with non-standardized clinical discharge criteria [79,80]. Some limitations should be acknowledged. These scoring systems are primarily applicable to outpatient procedures and do not include a formal assessment of the patient’s mental state or cognitive function. Therefore, evaluation of cognitive status should always be performed separately before discharge.

### 5.2. Patient Comfort

Patient comfort represents a fundamental component of GI endoscopy and is increasingly recognized as a key quality indicator that should be routinely assessed [81,82].

Evidence consistently supports the role of sedation in improving patient-reported outcomes during GI endoscopy. A large randomized controlled trial demonstrated that the use of sedation was associated with a higher adenoma detection rate, an increased cecal intubation rate, improved technical quality, and greater patient satisfaction, while maintaining a safety profile comparable to that of unsedated procedures [83].

In line with these findings, McQuaid et al., in a meta-analysis, reported that sedation significantly improved patient satisfaction (relative risk [RR] = 2.29, 95% range 1.16–4.53) and increased patients’ willingness to undergo repeat esophagogastroduodenoscopy (RR = 1.25, 95% range 1.13–1.38) compared with no sedation [84].

When different sedative regimens were compared, a multicenter randomized controlled trial in patients undergoing colonoscopy showed that propofol-based sedation resulted in higher patient satisfaction (86% vs. 74%), reduced procedure recall, and led to fewer complications compared with midazolam combined with fentanyl [85]. Consistently, two meta-analyses demonstrated greater patient satisfaction with propofol sedation than with traditional sedative agents [26,86].

However, a meta-analysis of three randomized controlled trials found no significant difference in patient satisfaction between moderate and deep sedation for colonoscopy, suggesting that deeper levels of sedation do not necessarily translate into improved patient experience [87].

Adequate sedation has been consistently shown to improve patient comfort, and the achievement of optimal comfort is increasingly considered a core parameter of high-quality sedation practice. Despite the validation of several assessment tools, robust evidence identifying the optimal instrument for evaluating patient comfort remains lacking. A recent observational study comparing the Gloucester Comfort Scale (GCS) with the Endoscopy Patient-Reported Experience Measure (EndoPREM) suggested that validated patient-reported experience measures may provide a more accurate assessment of patients’ colonoscopy experiences than the GCS, which is commonly used as the standard comfort metric [88]. Nevertheless, the routine implementation of EndoPREM may be constrained by the length of the questionnaire and the time required for its completion.

## 6. Training

**Figure 4:**
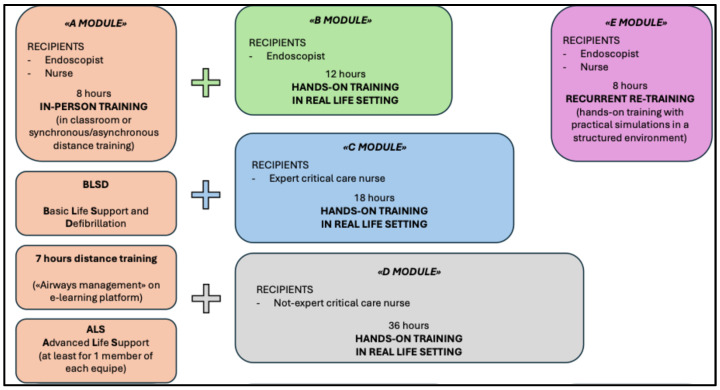
Synthesis of structured training program.

Sedation in GI endoscopy requires comprehensive and standardized training of the entire endoscopy team. ESGE and the European Society of Gastroenterology and Endoscopy Nurses and Associates (ESGENA) have defined a dedicated sedation curriculum outlining minimum requirements in terms of knowledge, technical skills, and clinical competencies [89]. The clinical effectiveness of structured sedation training has been demonstrated in large real-world cohorts. In a prospective study involving more than 12,000 GI endoscopic procedures, implementation of the ESGE–ESGENA training program was associated with a very low incidence of sedation-related adverse events, all of which were successfully managed by the endoscopy team without sentinel events [12]. Training programs should be considered mandatory for all professionals involved in endoscopic sedation, including endoscopists and nursing staff. Core components should include pharmacology of sedative and analgesic agents, patient selection and risk stratification, intraprocedural and postprocedural monitoring, and early recognition and management of sedation-related complications. Notably, a multicenter prospective Italian study highlighted substantial variability and lack of standardization in sedation practices across centers [10], leading to the establishment of a national task force that proposed a structured sedation training program, as illustrated in Figure 4. Overall, available evidence clearly supports structured sedation training as an essential pillar of high-quality and safe GI endoscopy and as a prerequisite for the implementation of modern sedation strategies.

## 7. Personalized Sedation

Patients undergoing GI endoscopy present with heterogeneous risks for sedation-related adverse events. Several patient- and procedure-related factors have been associated with an increased risk of hypoxemia, hypotension, airway obstruction, need for airway intervention, delayed recovery, or unplanned escalation of care. Patient-related risk factors include advanced age, frailty, higher ASA physical status, obesity, obstructive sleep apnea, significant cardiopulmonary disease, impaired functional status, and previous sedation-related adverse events. Procedure-related risk factors include prolonged procedure duration, complex therapeutic interventions, upper gastrointestinal or pancreatobiliary procedures requiring deeper sedation, non-supine positioning, and anticipated need for high-dose or deep sedation. Recognition of these factors is essential for pre-procedural risk stratification and for selecting an individualized sedation strategy [14,15,16,17].

Drug-centered sedation protocols, which primarily focus on the choice, dose, and combination of sedative agents, remain widely used but are inherently limited by their lack of flexibility and their tendency to apply standardized regimens across heterogeneous patient populations. Personalized sedation, by contrast, is a structured, patient-centered approach in which the sedation plan is selected according to a multidimensional assessment of patient-related risk, procedural complexity, intended depth of sedation, available monitoring, and local organizational resources. In this framework, sedation is not viewed solely as drug administration, but as a continuum of options ranging from unsedated or minimally sedated procedures to moderate or deep sedation, adjunctive techniques, enhanced monitoring, and anesthesiology-supported care when appropriate.

In our setting (Emilia-Romagna, Italy), an attempt has been made to operationalize the concept of personalized sedation by developing a structured, risk-adapted model. This approach is based on the combined assessment of patient-related risk factors and procedural complexity, allowing stratification into three levels of sedation with progressively increasing intensity.

**Figure 5:**
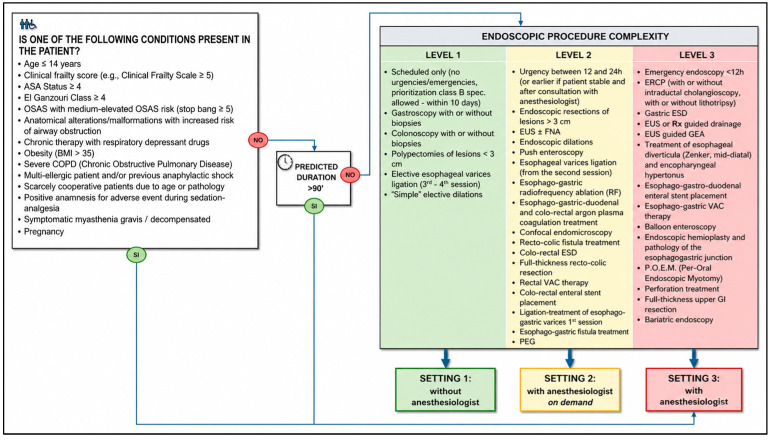
Proposed risk-adapted model for personalized sedation in gastrointestinal endoscopy. ASA, American Society of Anesthesiologists; BMI, Body Mass Index; ERCP, Endoscopic Retrograde Cholangiopancreatography; ESD, Endoscopic Submucosal Dissection; OSAS, Obstructive Sleep Apnea Syndrome; PEG, Percutaneous Endoscopic Gastrostomy; VAC, Vacuum-Assisted Closure.

This model aims to optimize the balance between safety, procedural efficacy, and resource allocation, providing a practical framework for implementing personalized, technology-supported sedation pathways in routine clinical practice (Figure 5). In practical terms, this approach can be applied through a stepwise pathway: first, identify patient-related risk factors; second, classify procedural complexity and anticipated duration; third, select the intended depth of sedation; fourth, match the monitoring strategy to the expected risk; and finally, define whether anesthesiology support is required before the procedure begins. Low-risk patients undergoing short diagnostic procedures may be suitable for unsedated, minimal, or moderate sedation pathways. Patients undergoing prolonged or therapeutic procedures, or those with relevant cardiopulmonary, frailty, obesity, or obstructive sleep apnea risk, may require deeper sedation, enhanced monitoring such as capnography, high-flow oxygen when appropriate, and/or anesthesiology involvement.

Nevertheless, the implementation of personalized, technology-supported sedation pathways presents several challenges. These include variability in resource availability, additional costs related to advanced technologies, the need for dedicated training, and organizational constraints that may limit their widespread adoption. Therefore, future efforts should focus not only on expanding the therapeutic and technological armamentarium, but also on ensuring its appropriate and sustainable integration into clinical practice, which ultimately relies on close and structured collaboration between endoscopists and anesthesiologists.

Although this narrative review does not aim to provide a guideline-by-guideline comparison, the proposed personalized pathway is aligned with core recommendations from ESGE, ASGE, and BSG guidance: pre-procedural risk assessment, titration of sedation depth to patient and procedural risk, careful use of propofol within trained teams, enhanced monitoring in selected higher-risk patients or prolonged procedures, and standardized post-procedural recovery and discharge criteria.

## 8. Future Direction

As sedation practices continue to evolve, several technological and pharmacological innovations are poised to further transform the field of GI endoscopy.

Among emerging technologies, AI represents one of the most promising tools. Machine learning and deep learning applications are expected to play an increasing role in the endoscopy environment, enhancing safety and optimizing peri-procedural management in the endoscopy suite, beyond the patient’s satisfaction [90]. Recent studies indicate that computer-aided diagnosis systems have substantial potential in supporting sedation quality control. Their integration into endoscopic practice has been associated with improved patient satisfaction, as well as reductions in both emergence time (from procedure completion to spontaneous eye opening) and overall recovery time [91]. In parallel, artificial neural networks are emerging as valuable tools for predicting hypoxemia during sedation for GI endoscopy. By incorporating multiple patient variables, such as body mass index, obstructive sleep apnea–hypopnea syndrome, habitual snoring, and neck circumference, these models can efficiently identify outpatients at higher risk for peri-procedural desaturation [92,93]. Limitations persist: AI cannot replace essential non-technical skills (e.g., emotional support) and current systems lack the integrative, human-like intelligence required to manage the full complexity of anesthesia care. For these reasons, the future of AI in endoscopy is likely to implement a collaborative model in which technology augments healthcare professionals.

An important future challenge in GI sedation is the identification of safe and effective pharmacological options especially for elderly and high-risk patients. Population aging and the increasing burden of comorbidities make it necessary to move toward more individualized, risk-adapted sedation strategies. In this context, emerging agents such as remimazolam and ciprofol have shown promising results, with growing evidence supporting efficacy comparable to propofol and a potentially improved respiratory and hemodynamic safety profile. While ongoing clinical research will help refine their optimal use, future efforts should also focus on the development and identification of sedative agents specifically designed to enhance safety in frail and high-risk populations, with the goal of expanding the therapeutic armamentarium beyond traditional approaches.

Traditionally, quality assessment in GI endoscopy has focused primarily on technical and diagnostic indicators, such as adenoma detection rate, cecal intubation rate, and procedure completion [94]. However, sedation represents an integral component of the endoscopic process and has a direct impact on patient safety, comfort, and procedural efficiency. Despite this central role, sedation-related outcomes have historically been underrepresented in quality frameworks [95]. The increasing adoption of digital endoscopy reporting platforms and advanced monitoring technologies facilitates automated acquisition of sedation-related metrics, reducing reporting burden and improving data reliability. Future research should focus on validating standardized sedation quality indicators and determining their association with clinical outcomes and healthcare resource utilization.

Environmental sustainability is increasingly recognized as a relevant aspect of healthcare delivery, including procedural sedation in GI endoscopy. In fact, sedation and in general anesthesia constitutes a critical domain with a huge potential to reduce the carbon footprint [96], even if little data exists on GI-related sedation. Sedation practices contribute to CO_2_ emissions through pharmaceutical waste, oxygen consumption, high-intensity resources such as operating rooms, or anesthesiology-led care. In a recent position statement, ESGE and ESGENA [97] recommend optimizing drug dosing, avoidance of unnecessary deep sedation, a saline fluid intravenous solution, inadequate antibiotic prophylaxis and the use of short-acting agents with rapid recovery. Recent evidence has demonstrated that intravenous anesthesia reduces the harmful effects of inhaled anesthetics, minimizing the use of nitrous oxide [96,98]. Further studies are needed to quantify the environmental impact of different sedation strategies in GI endoscopy, maintaining patient safety as a priority but incorporating sustainability considerations into sedation planning [97].

Future sedation pathways will likely shift from a drug-centered approach to an integrated, patient-tailored, and technology-supported model.

## 9. Conclusions

Sedation remains a cornerstone of modern GI endoscopy, enhancing both patient comfort and procedural quality. Over the next decade, sedation in GI is expected to evolve toward an integrated, patient-centered, and technology-supported model, in which pharmacological innovation, advanced monitoring, and structured training will converge to optimize safety and quality of care.

## Figures and Tables

**Table 1:** Main characteristics of the pharmacological agents.

Name	Onset	Effect Duration	Advantages	Limitations
Fentanyl	60–120 s	30–60 min	Potent analgesia, rapid onset, hemodynamic stability, reversible with naloxone	Respiratory depression, chest wall rigidity, accumulation with repeated doses
Midazolam	120–240 s	30–120 min	Anxiolysis, amnesia, anticonvulsant properties, reversible with flumazenil	Prolonged recovery, respiratory depression, accumulation (especially in elderly)
Remimazolam	60–120 s	5–10 min	Rapid onset/offset, organ-independent metabolism, reversible with flumazenil	Higher cost, limited clinical experience
Propofol	30–60 s	3–15 min	Very rapid onset and recovery, easy titration, antiemetic	Hypotension, respiratory depression, injection pain, lack of reversal agent
Ciprofol	30–60 s	5–10 min	Similar to propofol, less injection pain	Limited data, potential cardio-respiratory depression, lack of reversal agent
Dexmedetomidine	180–600 s	15–45 min	Cooperative sedation, minimal respiratory depression, some analgesia	Bradycardia, slower onset

**Table 2:** General characteristics of meta-analyses comparing remimazolam with propofol.

First Author (Year)	N° of Studies (RCTs)/N° of Patients	Procedures	Outcomes in Experimental Group (Remimazolam)	Similar Outcomes/Outcomes in Control Group (Propofol)
Wang R (2025) [39]	17/4525	GI endoscopy	Lower respiratory depression, injection pain, hypotension, hypoxemia	Recovery time/improved sedation success, longer sedation time
Li FZ (2024) [40]	7/1445	GI endoscopy	Lower hypotension, respiratory depression, injection pain, bradycardia, shorter discharge times	Postoperative nausea and vomiting, dizziness, successful sedation rate, time to become fully alert
Terres MT (2024) [41]	7/1499	GI endoscopy	Lower risk of adverse events, including hypoxemia, respiratory depression, hypotension, bradycardia, and injection pain	Postoperative nausea and vomiting, dizziness and headache
Ahmer W (2024) [42]	7/1466	GI endoscopy	Lower risk of bradycardia, hypoxemia and pain on injection site	Sedation time, number of supplemental doses, procedural parameters/lower time to loss of consciousness, greater sedation success after 1° dose
Barbosa EC (2024) [43]	15/4516	GI endoscopy	Lower sedation success rate, slightly longer induction time, lower rates of respiratory depression, hypotension, bradycardia	Patient and endoscopist satisfaction
Kim J (2025) [44]	5/965	ERCP	Lower hypoxia, hypotension, and bradycardia	Completion rate and risk of delirium or agitation

GI, gastrointestinal; ERCP, endoscopic retrograde cholangiopancreatography; RCT, randomized controlled trial.

**Table 3:** General characteristics of meta-analyses comparing ciprofol with propofol.

First Author (Year)	N° of Studies (RCTs)/N° of Patients	Procedures	Outcomes in Experimental Group (Ciprofol)	Similar Outcomes/Outcomes in Control Group (Propofol)
Cheng X (2025) [46]	17/2800	GI endoscopy	Longer induction time in patients under 65 years old, lower hypotension, bradycardia, injection pain, respiratory depression, hypoxemia	Sedation success rate or recovery time
Yu Y (2025) [47]	9/1860	GI endoscopy	Lower hypotension, respiratory depression, hypoxemia, choking cough, injection pain	Bradycardia, involuntary movement, dizziness, nausea, and vomiting
Yang H (2025) [48]	45/6884	GI endoscopy	Lower hypotension, bradycardia, nausea and vomiting, hypoxia, respiratory depression, apnea, injection pain, higher satisfaction among patients and anesthesiologists	Procedural efficiency
Liu J (2024) [49]	10/1545	GI endoscopy	Lower injection pain	Sedation efficacy
Ortegal GH (2024) [50]	6/1225	GI endoscopy	Lower risk of respiratory depression, injection pain, significantly higher patient satisfaction	No significant differences in other adverse events, time-related outcomes, probability of procedure success
Wang Z (2025) [51]	20/3779	GI endoscopy	Lower injection pain, hypotension, bradycardia, overall respiratory disorders, shorter time to onset of successful induction, longer discharge time	Successful rate of sedation and waking time
Wu K (2025) [52]	7/1264	ERCP	Lower bradycardia, hypotension, respiratory depression, hypoxemia, injection pain	Choking cough, involuntary movements, or nausea and vomiting

GI, gastrointestinal; ERCP, endoscopic retrograde cholangiopancreatography; RCT, randomized controlled trial.

## Data Availability

No new data were created or analyzed in this study. Data sharing is not applicable to this article.

## Abbreviations

The following abbreviations are used in this manuscript:

AI	Artificial intelligence
BIS	Bispectral index
ESGE	European Society of Gastrointestinal Endoscopy
ESGENA	European Society of Gastroenterology and Endoscopy Nurses and Associates
ERCP	Endoscopic retrograde cholangiopancreatography
GI	Gastrointestinal
HFNC	High-flow nasal cannula
mPADDS	modified Post-Anesthetic Discharge Scoring System
RCT	Randomized controlled trial
